# The Large-Scale Deployment of Fish Aggregation Devices Alters Environmentally-Based Migratory Behavior of Skipjack Tuna in the Western Pacific Ocean

**DOI:** 10.1371/journal.pone.0098226

**Published:** 2014-05-21

**Authors:** Xuefang Wang, Yong Chen, Samuel Truesdell, Liuxiong Xu, Jie Cao, Wenjiang Guan

**Affiliations:** 1 College of Marine Sciences, Shanghai Ocean University, Shanghai, China; 2 National Engineering Research Center for Oceanic Fisheries, Shanghai, China; 3 The Key Laboratory of Sustainable Exploitation of Oceanic Fisheries Resources, Ministry of Education, Shanghai, China; 4 School of Marine Sciences, University of Maine, Orono, Maine, United States of America; North Carolina State University, United States of America

## Abstract

Fish aggregation devices (FADs) have been used extensively in the tuna purse seine fishery since the 1980s. This long-term modification of natural habitat has generated discussions as to whether FADs impact movement patterns of tuna species. We examined this question using data collected from the skipjack tuna (*Katsuwonus pelamis*) fishery. We used the longitudinal gravitational center of catch (*G*) to examine temporal variability in skipjack movement in the Western and Central Pacific Ocean, and related this to El Niño Southern Oscillation (ENSO) events. We found that in most cases *G* for free-swimming school sets changed with the onset of ENSO events, while *G* for floating-object-associated school sets remained relatively constant. This suggests that skipjack exhibit distinguishable behavioral strategies in response to ENSO events: they either react by moving long distances or they associate with floating objects. There has been no previous attempt to evaluate the interaction between FADs and the environmentally-determined movement of skipjack; this study shows evidence of an interaction, which should be considered when managing skipjack populations.

## Introduction

The tuna purse seine fishery, which accounts for much of the global tuna catch, is divided into two parts: effort that targets free-swimming schools and effort that targets aggregations associated with floating objects. The attractive nature of floating objects for tuna and tuna-like species greatly increases their catchability, and since the presence of natural floating objects is not always predictable, fishermen often construct and deploy artificial fish aggregation devices (FADs) and have done so since the 1980s [1], [2]. This fishing method expanded rapidly during the 1990s, and currently 40% of the global tropical tuna catch comes from purse seine sets on floating objects [3], despite that FAD-associated catches of bigeye (*Thunnus obesus*) and yellowfin (*Thunnus albacares*) tuna are comprised mainly of smaller fish [4].

There has been considerable discussion among fisheries scientists over the potential impacts of the large scale deployment of FADs on tuna stocks, particularly impacts that are ecological in nature; for instance whether FADs might alter the natural movements of tunas [5], [6]. It has been suggested that high concentrations of FADs may even function as an “ecological trap” for tunas [5]: FADs could entrain tunas in unsuitable locations, changing stock condition as well as the spatio-temporal dynamics of the population. Evidence has been presented that demonstrates significant differences in many biological and ecological characteristics of tunas associated with floating objects as opposed to those in free-swimming schools; e.g., feeding patterns [6], [7], [8], fish condition [5], [6], growth rates [5], [6], aggregation patterns [9], and migratory direction and displacement rates [6]. However, there is still a limited understanding of the potential long-term effects of FADs on tuna stocks and pelagic ecosystems [1], [10].

The Western and Central Pacific Ocean (WCPO) has unique oceanographic patterns that include warm-cool temperature boundaries that attract tuna; this area contributes over 55% of the global tuna catch [2]. Using catch per unit effort (CPUE) data as a proxy for the abundance of fish stocks, Lehodey et al. [11] found that skipjack tuna (*Katsuwonus pelamis*) appear to follow the 29°C sea surface temperature isotherm, which represents the temperature convergence zone on the eastern edge of the western Pacific warm pool. Considerable longitudinal displacements of this isotherm occur in phase with the El Niño Southern Oscillation (ENSO) cycle, i.e., the southern oscillation index (SOI), and skipjack engage in corresponding movements to follow this edge.

While this previous research has shown how climatic forcing can drive movement in free-swimming schools, this study expands on the characterization of movement patterns by clarifying the interaction between the distribution of the skipjack fleet, ENSO and FADs. Since fishery-independent data are unavailable, we assume here that the spatial distribution of the catch mirrors that of the population; i.e., fishermen follow an ideal free distribution [12] with respect to skipjack. Similar catch-based assumptions have been made in other research on tuna and tuna-like species [11], [13]. This research is important because understanding movement patterns is essential for the effective management of highly migratory species such as skipjack tuna.

## Methods

Purse seiners employ different techniques for free-swimming schools and floating object-associated aggregations. Object-associated sets typically occur before sunrise and seiners usually set only once per day, while they often set many times per day on free-swimming schools and at any time. The success rates differ as well: while sets around free-swimming schools succeed about 50% of the time, associated sets have an approximately 90% success rate [14]. A single day of purse-seining might be comprised of sets on both types of schools. The differences in success rate between the two types of fishing and the fact that boats often engage in both associated and free-swimming sets in a single day make the use of effort incomparable between the school types, so we focus our analyses on catch alone.

In examining the relationship between the gravitational center of skipjack stocks and ENSO events, Lehodey et al. [11] used the longitudinal gravitational center of CPUE in the United States purse seine fishery from 1988–1995 as a spatial index of highest fish stock abundance to explore the relationship between the abundance center of free school catch-per-unit-effort and the ENSO cycle. Up until 1996, covering the period of Lehodey's study, more than 80% of sets the purse seine fleet were on free-swimming schools; after 1996 FAD fishing became more common [15].

In this study, we used the combined catch data from 1991 to 2005 for purse seine fleets operating in the Western and Central Pacific Ocean, which includes sets on free swimming and object-associated aggregations. Unlike in Lehodey's data, which could be standardized by effort, during this period effort has shifted towards FAD aggregations and there are considerable differences in fleet size, vessel performance, fishing technology and set type preference among these fleets [15]. These factors make any calculation of fishing effort (and thus CPUE) likely to be misleading (effort would appear accounted for but it is unlikely to be accurate). As such, we decided to use the gravitational center of catch directly instead of the center of CPUE (as ref. 11 did).

Purse seiners actively search for fish schools to set on, and employ advanced technology such as modern sonar, helicopters, data from buoys and satellites and fish forecasting models, allowing vessels to find both free-swimming and FAD-associated tuna aggregations efficiently. Given the technology used and the absence of other metrics, we assume that the catch locations sufficiently represent the distribution of the population.

The longitudinal gravitational center of catch (i.e., the mean longitude weighted by catch) by month (*G_m_*) in our study was defined as:
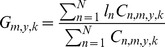
where *l* is longitude, *m* is month, *y* is year, *k* is the type of fishing effort, and *C_n,m,y,k_* is the skipjack catch at longitude *n* in month *m* in year *y* and for fishing type *k*. Similarly, the seasonal longitudinal gravitational center of catch (*G_s_*) was calculated with seasonal catch data instead of monthly catch data, so catch is summed over season instead. Seasons were three-month periods beginning with January-March 1991.

The tuna purse seine catch data used in this study came from the Western and Central Pacific Fisheries Commission (WCPFC) database, which includes monthly average landings by fishing method (floating object-associated or free-swimming school) and by year and month. The data cover the Western and Central Pacific Ocean, aggregated in 5° latitude ×5° longitude squares.

The SOI indicates the intensity of El Niño or La Niña events in the Pacific Ocean. We obtained SOI records from the University of East Anglia Climate Research Center and considered La Niña events to occur when the SOI exceeded 1.0 and El Niño when it was less than −1.0.

## Results

**Figure 1:**
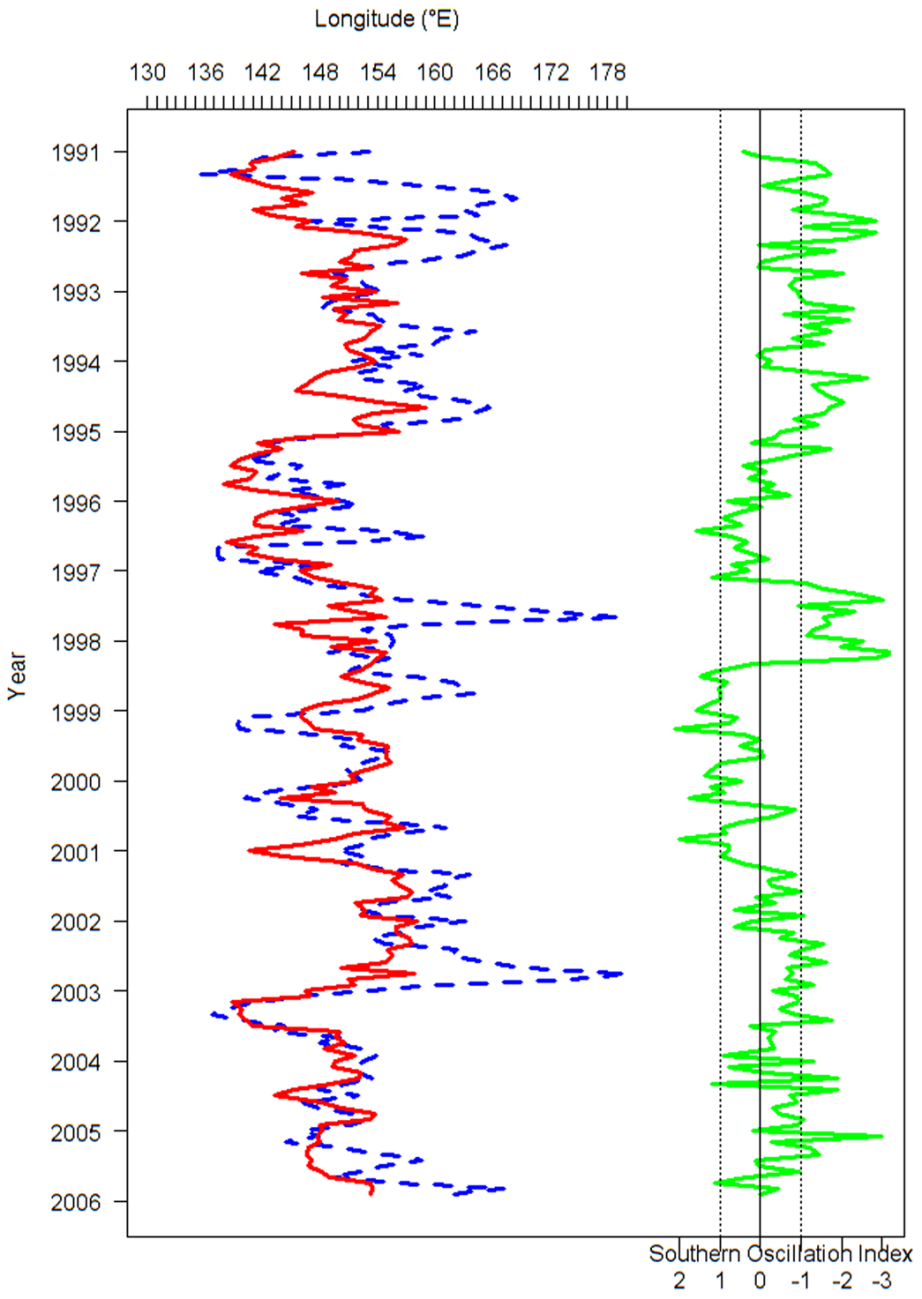
The longitudinal gravitational center of catch by month (*G_m_*) for free-swimming and drifting-floating-object-associated school sets of skipjack tuna and the southern oscillation index (SOI). The blue line represents free-swimming catch, the red line represents drifting-floating-object-associated catch, and the green line is SOI.

**Figure 2:**
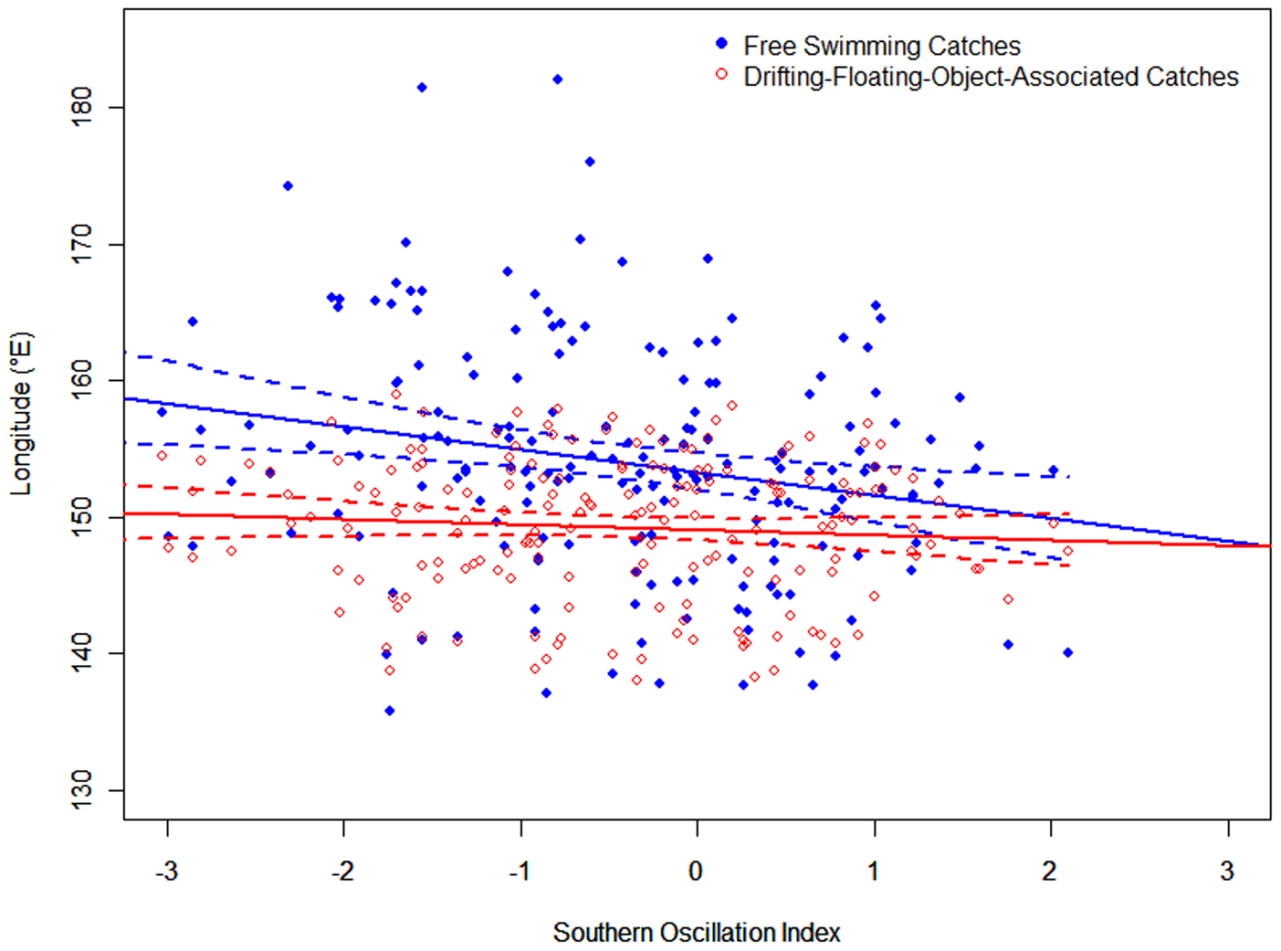
Scatter diagram of the longitudinal gravitational center of catch by month (*G_m_*) free swimming school and drifting-floating-object-associated school sets of skipjack tuna and southern oscillation index (SOI). The blue points represent free-swimming catch, the red points represent drifting-floating-object-associated catch. The blue and red solid lines are regression lines with dashed 95% confidence intervals.

We calculated the monthly longitudinal gravitational center of catch (*G_m_*) for the free-swimming school sets and floating object-associated aggregation sets of skipjack from 1991–2005, and we compared *G_m_* with the SOI. The *G_m_* for free-swimming school sets had a similar pattern to the corresponding monthly SOI (Fig.1) with eastward displacement occurring during El Niño episodes and westward displacement during La Niña episodes. In contrast, *G_m_* for floating object-associated aggregation sets does not fluctuate as dramatically with phases of the ENSO cycle. *G_m_* for free-swimming school sets showed a general trend that shifted from east to west as the SOI increased, while *G_m_* for floating object-associated aggregation sets always fluctuated within a relatively constant range regardless of the SOI (Fig. 2). A paired t test indicated that there was a significant difference in the *G_m_* between free-swimming and associated aggregation sets (*t* = 9.05, *p*<0.001).

**Figure 3:**
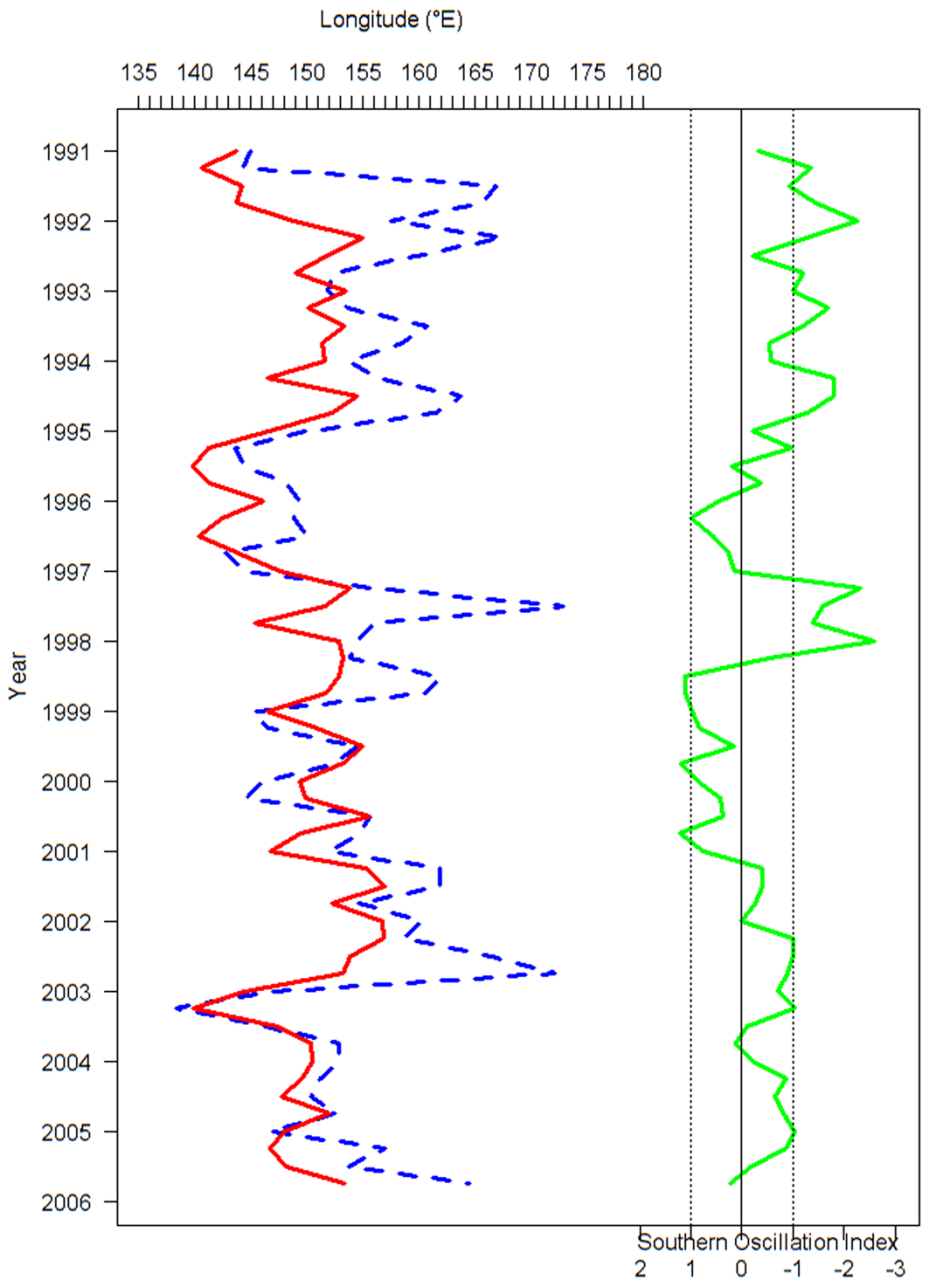
The longitudinal gravitational center of catch in season (*G_s_*) for free-swimming and drifting-floating-object-associated school sets of skipjack tuna and the southern oscillation index (SOI). The blue line represents free-swimming catch, the red line represents drifting-floating-object-associated catch, and the green line is SOI.

The correspondence between the peaks in *G* and the SOI is more apparent when the gravitational center of catch is aggregated by season (*G_s_*). When aggregated by month (*G_m_*) the data are noisier and the relationship less clear (Figs. 1 and 3).

*G_s_* for free-swimming school sets shifted to the east in 1991–1993, 1994–1995, 1997–1998, 2002–2003, and most noticeably in 1991–1992 and 1997–1998 when strong El Niño events occurred. In these years, the difference in *G_s_* between the two types of fishing sets was over 20° of longitude (Fig. 3). A paired t test indicated that there was a significant difference in the seasonal gravitational catch centers (*G_s_*) between the two types of fishing (*t* = 6.24, *p*<0.001).

**Figure 4:**
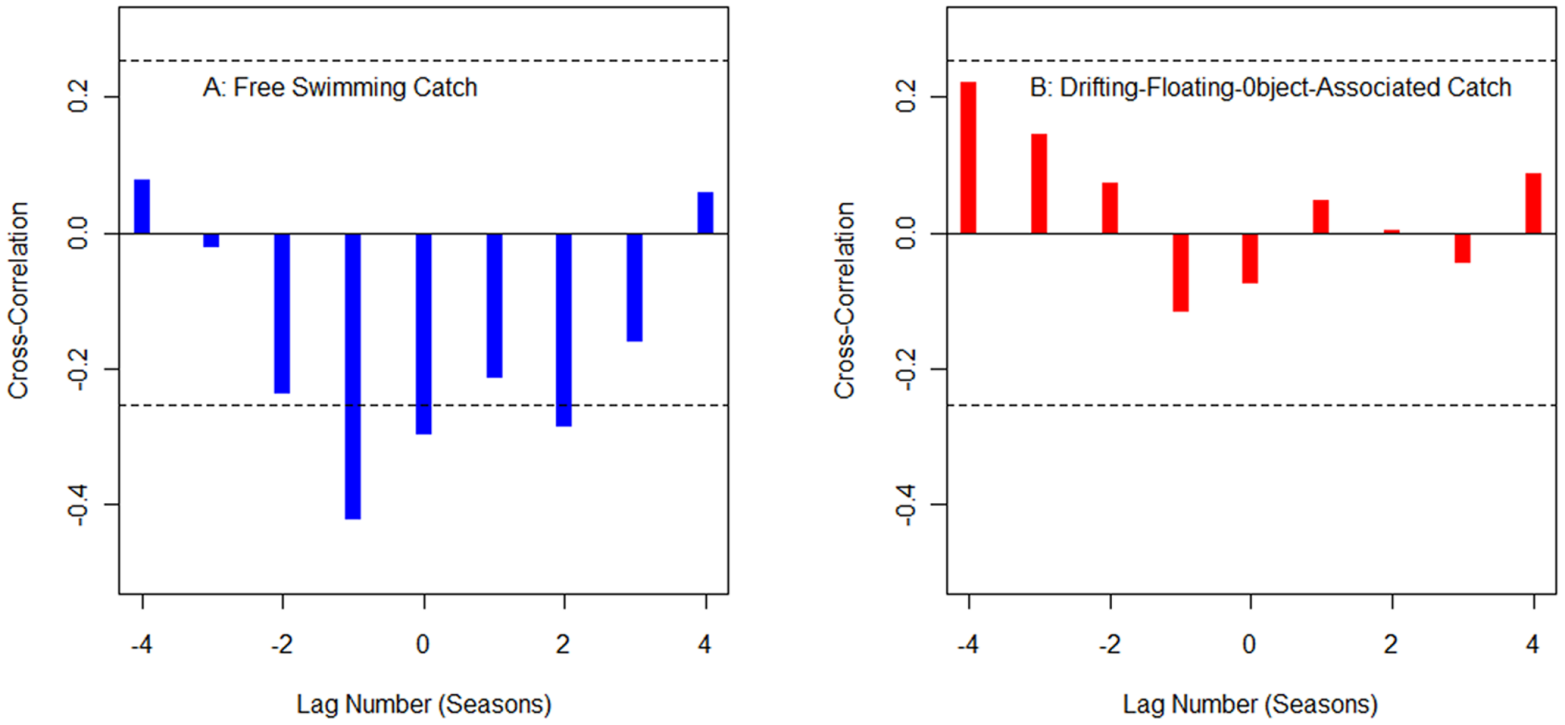
Correlation coefficients between the longitudinal gravitational center of catch by season (*G_s_*) and the southern oscillation index (SOI). A: free-swimming catch; B: drifting-floating-object-associated catch. The lag metric for horizontal axis is season; the dash line represents the correlation 95% confidence intervals which are unadjusted for multiple comparisons.

*G_s_* for the free-swimming school sets was correlated with the SOI (Pearson's *R* = −0.423; *p*<0.05; *p*-value unadjusted for multiple comparisons of tests at different lags) at a lag of −1 season (Fig.4). A lag of −1 is reasonable because it takes time for the effects of an El Niño event to be reflected in the behavior of fish and fishermen. The maximum correlation for associated aggregation sets with the SOI was 0.22 at a lag of −4 seasons, but this was not statistically significant (Fig.4, *p*>0.05).

**Figure 5:**
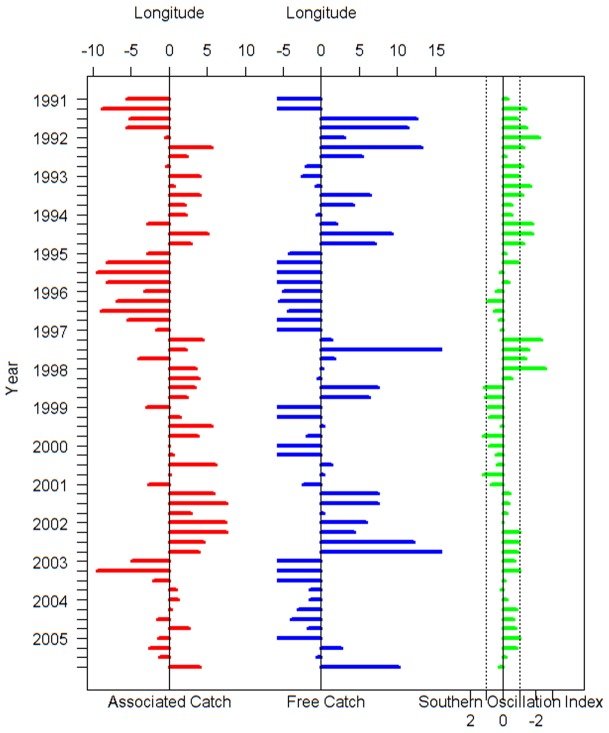
Anomalies for the longitudinal gravitational center of catch by season (*G_s_*) for free-swimming and drifting-floating-object-associated school sets of skipjack tuna and the southern oscillation index (SOI).

Finally, *G_s_* anomalies for the two types of fishing also suggested that the seasonal variations in *G_s_* for associated aggregation sets are smaller than those of free school sets during El Niño episodes such as1991–1993, 1997–1998 and 2002–2003 and La Niña episodes such as 1999–2001 (Fig.5).

## Discussion

### Differences in *G* by school type

Our study reveals an important interaction between the spatial distribution of skipjack catch, FADs and ENSO. When the ecosystem is less affected by SOI climate forcing, the catch of free-swimming and associated skipjack occurs within a similar longitudinal range; however, a dramatic difference in the longitudinal position of these two catch types appears when ENSO alters the environment (Figs. 3 and 5). This study supports previous research (e.g., ref. 11) by showing (assuming catch to be a good proxy for abundance) that some skipjack respond to ENSO conditions by moving great distances. However, these analyses evidence that this behavior is not universal, and some skipjack exhibit an alternate strategy of exploiting floating object habitat instead.

We assume the change in the spatial distribution of fishing effort observed in this study to be the result of ENSO-driven movement of free-swimming schools. We cannot offer an alternative explanation for why the spatial distribution of fishing effort targeting skipjack would change dramatically other than the fishery following the resource, but it should be reiterated that our inferences regarding the behavior of skipjack are made using catch data alone. Large-scale movement of skipjack may be attributable to the presence of an oceanic convergence front which is likely a feeding area [16], [17]; consequently the displacement of the convergence by ENSO drives the large scale dispersal of free schools [11]. Distance movements by skipjack are an adaptation to an environment with dynamic, heterogeneous prey fields and where large-scale change is common [18]. The ability of free-swimming schools to migrate with large-scale oceanographic fronts is not obvious over short time scales with little contrast, but becomes evident in longer time series that encompass significant environmental change.

It should be noted that while we found the gravitational center of catch to shift with ENSO, this does not imply that skipjack completely vacate certain areas, or even that all skipjack are affected by ENSO. The gravitational center of catch represents only a tendency for the center of the catch distribution to shift.

By contrast, the catch of FAD-associated aggregations is determined by the FAD deployment strategies of fishermen. Their tendencies are influenced by both ecological and socioeconomic factors, such as the cost of fuel, oceanographic features, the potential poaching rate of an area, economic circumstances, etc. [19]. In the WCPO, drifting FADs are more commonly deployed east of 160^°^E, and natural floating objects distributed mostly west of 170° E in the waters off larger land masses [2]; consequently the gravitational centers of catch for associated aggregation sets generally remain within this region regardless of the SOI (Figs 3 and 5).

### Inconsistencies between *G* and SOI

The displacement of *G_m_* or *G_s_* for free swimming school sets does not always achieve a perfect match with the variation in SOI. For example, given the typical trends we saw in the *G_s_* for free-swimming school sets, early in 2001 the catch was farther to the east than expected given the La Ninã event in late 2000. Similarly, in late 2005 there was no El Niño event, but the *G_s_* of free-swimming school sets was located further east than would be expected (Fig.3).

Lehodey et al. suggest that an imperfect relationship between the *G_s_* peak locations for free-swimming school sets and the SOI may result from: (*i*) fishermen's behavior and a breakdown of the relationship between fishing effort and skipjack abundance; (*ii*) natural variability in the relationship between skipjack and the convergence zone; or (*iii*) a combination of these two processes [11].

We believe that the catch data aggregated on a 5°×5° grid has captured the overall trend relating the longitudinal gravitational centers for the two set types and the SOI, although this trend or correlation may be more obvious if a finer spatial resolution (e.g., 1°×1°) was available.

### FADs, ENSO and skipjack behavior

Previous research that used telemetry to track tuna indicates that the presence of FADs has no effect on the large-scale movement patterns of individual bigeye tuna [20] or that the tendency of yellowfin and bigeye tuna to leave an anchored FAD area was not significantly altered by a high density of FADs [21]. However, the analyses presented here imply that on a population scale FADs have the potential to change the movement patterns of skipjack; during ENSO events, when some of the fishery is displaced great distances (presumably following the tuna) there is still a consistent associated catch. This fact suggests multiple behavioral strategies for skipjack to cope with environmental change; one strategy being movement and the other associating with (or remaining associated with) floating objects.

Given the evidence that skipjack use floating objects as an alternative strategy to large-scale movement, it is important to consider what effect high densities of floating objects may have on the population. The number of FADs has increased considerably, and they are often deployed in areas that lack natural floating structure, for instance in the WCPO fishing grounds [2].This increase in the number of artificial floating objects may impact the population-scale behavior of tuna stocks.

Given the nature of the data used in these analyses, it is impossible to rule out an alternative hypothesis: FADs do not alter skipjack behavior because the two behavioral modes exist regardless of FAD presence. During an ENSO event, or whenever conditions become less favorable some fish will move long distances while others remain. However, tagging studies have shown that the same fish that are attracted to FADs are also capable of long-distance movements [20]. This suggests that the same skipjack can use multiple strategies for finding an appropriate habitat. As such, we assert that the habitat provided by FADs may represent an alternative strategy for coping with changing environmental conditions. In order to explicitly test the impact of FADs on skipjack fitness, however, it would be necessary to compare the condition of free schooling and FAD-associated skipjack in both El Nino and non-El Nino years.

### Application to management

FADs are known to impact tuna populations because they increase vulnerability to fishing, but research has also suggested that FADs may also cause population-level changes that go beyond the direct fishing effects by altering the relationship between tuna and their environment [22]. Our research offers support to this argument, given that FADs appear to offer skipjack an alternative strategy to large-scale movement. FADs are an example of the indirect effects of fishing, which has been shown to impact fish populations through, for instance, physical habitat alteration [23], changing community structure [24] and selective pressure on different phenotypes [25].

While current policies do include regulations that address FAD fishing, many scientists believe that the use of FADs needs to be more tightly controlled given their impacts on tuna populations [10]. Many FAD management scenarios have been considered and implemented by regional fisheries management organizations, such as time and area closures [10], [26], prohibiting sets on FADs [27], restrictions on number of sets on FADs [28], restrictions on number of FADs per vessel [29], and bans on discards [28], [30].

The intent of these catch and effort controls is to reduce fishing mortality on juvenile tuna and by-catch associated with FADs, and the potential non-vulnerability-related effects are seldom considered. For example, techniques such as time and area closures or prohibiting sets on FADs can reduce overall fishing effort on FADs but do not require the mandatory removal of FADs from the water [27], so any ecological impacts remain intact. This is not to say that impacts necessarily decrease fitness; that would require data on skipjack condition. However, our data do show that, for better or worse, FADs are likely to play a role in the interaction between skipjack and their environment, thus altering the natural state of the population, and as such the ecological impacts beyond simply increasing the vulnerability of tunas ought to be considered by managers.
